# Nutritional Quality and Degree of Processing of Children’s Foods Assessment on the French Market

**DOI:** 10.3390/nu14010171

**Published:** 2021-12-30

**Authors:** Celine Richonnet, Françoise Mosser, Elisabeth Favre, Martine Robert, Françoise Martin, Isabelle Thiebaut

**Affiliations:** Club Européen des Diététiciens de l’Enfance (CEDE), Esplanade, 17-7800 Ath, Belgium

**Keywords:** children, child-oriented food, food marketing, HFSS, nutritional quality, Nutri-Score, food composition, nutrition labelling, sugar, ultra-processed foods

## Abstract

Food packaging marketing techniques which appeal to children (such as cartoon characters and brand mascots) affect children’s choices, preferences, and eating habits. Several studies have assessed the nutritional quality of food intended to children in various countries and concluded that most were high in fat, salt, and sugar (HFSS) and ultra-processed foods. The aim of this study is to analyse products intended for children over the age of 3 (foods and beverages with relevant marketing elements on the packaging) available on the French market as regards: (1) nutritional quality, based on the Nutri-Score labelling system, (2) compliance with expected nutritional profile suitable for children, according to the criteria of the WHO Europe Nutrient Profile Model, and (3) degree of processing, as defined by the NOVA classification, from packaging collected in 20 stores (hyper/supermarkets, hard-discount retail chains, and organic food stores). The marketing strategies most often used on children’s products are cartoons (97.22%; *n* = 1120) and mascots (77.78%; *n* = 896). A total of 1155 products were included in the study, most of which were sugary foods: almost a quarter of the products in the sample (23.81%; *n* = 275) list a sweetener as the first ingredient, and most of them (89.52%; *n* = 1034) contain free sugars according to the WHO definition. All the products included in our study feature marketing elements targeting on the packaging, yet 94.88% do not meet the criteria of the WHO Europe Nutrient Profile Model. Most (58.68%; *n* = 676) belong to Nutri-Score groups D and E, with the highest proportion in group D (39.32%; *n* = 453) and are ultra-processed (87.97%; *n* = 1016), especially through the use of flavourings and ultra-processed sugars. Using the Nutri-Score, the WHO Europe Nutrient Profile Model, and the NOVA classification, this study suggests that a significant share of pre-packaged foods marketed to children do not have an adequate nutritional profile. As such, measures are needed to regulate what marketing elements aimed at children can be included on packaging, based on these criteria.

## 1. Introduction

Among the various factors linked to overweight, obesity, and diet-related diseases, advertisements for foods high in fat, salt, and sugar (HFSS) have been shown to have a negative impact, particularly among children [1,2,3,4,5,6]. Marketing methods that target children, such as cartoon characters, brand mascots, toys, games, and promotions [7,8], affect the food preferences of children under 12 years old [5,9], the food prescription (pressure on parents), and are associated with a higher weight in children and adolescents [2,6,10,11]. When such marketing elements are included on packaging, they provide a competitive advantage, create brand differentiators, make products more memorable [12], and even improve perceived taste [13].

According to a report by the European Commission, 64% of online advertisements and 75% of TV spots for food and beverages shown to children in Europe in 2017–2019 were for HFSS foods [14]. In France, 70.0% of products advertised on TV to 4- to 12-year-olds in 2018 should not be shown to children according to the criteria of the WHO Europe Nutrient Profile Model [15] and 53.3% were classified Nutri-Score groups D and E [11,16]. Moreover, advertisements targeting children mainly promote ultra-processed foods (UPFs) according to observations made around schools [17,18,19] and children’s TV shows (98.9% of spots for UPFs in Argentina [20]). For this reason, many high-level general policy documents call for reducing the pressure of food advertising on children [6,21].

Several studies have assessed the nutritional quality of children’s food products that feature such marketing elements, including in the United Kingdom [22,23] and Australia [24,25]. In France, several consumer guides have assessed processed foods intended for children, in particular via the Nutri-Score labelling system [26,27], but they do not always specify the method of selection and they often mix products with and without marketing targeting children.

The aim of this study is to analyse the nutritional quality of products marketed to children over the age of 3 (i.e., food and beverages whose packaging shows marketing elements targeting children) available on the French market, taking the following aspects into account:

(1) nutritional quality, based on the Nutri-Score labelling system;
(2) compliance with expected nutritional profile suitable for children, according to the criteria of the WHO Europe Nutrient Profile Model;
(3) degree of processing, as defined by the NOVA classification.

The analysis was performed based on labelling on packaging found in stores (hypermarkets and supermarkets, hard-discount (HD) retail chains, and organic food stores) in order to accurately reflect the products available on the French market over the given period.

## 2. Materials and Methods

### 2.1. Data Collection

Information was collected from packaging on food products available in stores (hypermarkets and supermarkets, hard-discount retail chains, and organic food stores) between 23 October 2021 and 7 January 2021. The following 20 stores were included because they make up 98.7% of the value market share of hypermarkets, supermarkets and hard-discount retail chains cumulated for all of 2020 according to the French business and consumer magazine LSA: Aldi, Auchan, Biocoop, BioMonde, Carrefour, Casino, Colryut, Cora, Franprix, Intermarché, La Vie Claire, Leader Price, L’eau Vive, Leclerc, Lidl, Monoprix, Naturalia, Naturéo, O’bio, and U.

All food and beverages with at least one marketing element targeting children on the packaging were included, from all aisles except the baby food aisle. The elements were listed from publications about children’s exposure to food marketing [14,22,23,24] and from the protocol of the International Network for Food and Obesity/NCDs Research Monitoring and Action Support (INFORMAS) [28]. The definition used includes childlike drawings; mascots (cartoon characters, brand mascots, superheroes, personified food products); licenses (e.g., Barbie); childish names, funny names or fonts; games on the packaging or encouragement to play an online game that appeals to children’s cognitive abilities; primes (physical or virtual gifts for children); use of “tu”; drawings or text relating to children or their characteristics (rucksack, skateboard, hopscotch, etc.) such as “the perfect snack for kids”.

This store check was deemed preferable to collecting information from online databases, whether for professionals (CodeOnline Food) or consumers (Open Food Facts), which contain transcription errors, missing items, and outdated products, in order to accurately reflect the products available on the market over the given period.

The products were systematically picked up by an in-store operator and were not recorded twice if they were encountered more than once. For example, a national brand product found in several different stores was recorded only once: when it was first encountered, regardless of the store type (hypermarket/supermarket or hard-discount retail chain). As such, it is impossible make reliable comparisons between information collected at different stores or between the stores themselves. Different formats of the same product were not recorded to ensure that a recipe was analysed only once. Any duplicates were removed.

All packaging was photographed in its entirety and all useful data were processed, including the product name, the brand name, the EAN, the legal name, marketing elements aimed at children, ingredient list, nutritional values, nutrition claims, health claims, ingredient claims, references to children, Nutri-Score label, Nutri-Score result, organic label, and recommended portion size. Any datum recorded manually by one of the authors was checked by a different operator. Any errors found (which never exceeded 10% of the sample) were re-verified before being imputed.

### 2.2. Data Analysis

#### 2.2.1. Nutri-Score

The nutritional quality of the foods was established using Nutri-Score. Since the French Decree of 31 October 2017 was adopted [29], food manufacturers and distributors can use a new label that displays the product’s Nutri-Score group. Nutri-Score is a front-of-pack nutrition label that translates the nutritional quality of a product into a five-letter code (A, B, C, D, and E), each letter corresponding to a different colour. Energy, total sugar, saturated fat, and sodium score negative points, while fruits, vegetables, legumes, nuts, oils (rapeseed, nut, and olive), protein, and fibre score positive points. The algorithm on which Nutri-Score is based is on a continuous and discrete scale ranging from +40 (least healthy) to −15 (most healthy). The total sum of the score is divided into five groups (group A includes scores between −15 and −1, group B includes scores between 0 and 2, group C includes scores between 3 and 10, group D includes scores between 11 and 18, and group E includes scores between 19 and 40).

The French National Nutrition and Health Program (PNNS 4) 2019−2023 recommends using the Nutri-Score to choose the foods that should be encouraged, and to reduce the consumption of products in Nutri-Score groups D and E, especially in children [30].

We calculated the Nutri-Score for 1152 products (1155 products recorded, excluding 3 food supplements not subject to nutrient labelling), with or without Nutri-Score label, by analysing the information provided on the packaging, after determining the category, using the official table providing by Santé Publique France (the French National Health Agency) [31].

#### 2.2.2. WHO Europe Nutrient Profile Model

In 2015, the Regional Office for Europe of the World Health Organization (WHO) developed the Nutrient Profile Model [15] in order to monitor food marketing and advertising targeting children. The model can be considered a reference as regards nutrition and the composition of children’s food. It contains 17 food categories for which thresholds have been set, taking into account the nutrients that should be limited depending on how relevant they are for the category in question: energy, total fat, saturated fatty acids, total sugar, added sugars, sweeteners, and salt. Non-compliance (intake above the threshold) with regard to a single nutrient makes the food or beverage unsuitable for being advertised to children. In the case of certain foods (chocolate, sweets/candy, spreads, cereal bars, biscuits, cakes, pastries, fruit juices, energy drinks, ice cream, etc.), no nutritional criteria were set because such products are banned from advertising aimed at children. The products analysed were therefore categorised according to the profiling system nomenclature, which meant for example that some “juices and nectars” (with added sugar, water, vitamin C, or citric acid) had to be included in the “other beverages” category, which is different from the 100% fruit “juice” category.

#### 2.2.3. Sugar Intake Recommended in France

High consumption of sugars has been linked with dental caries and increased body weight from early in life till later in adulthood [32]. The upper limits for total sugars (mono- and disaccharides) excluding lactose and galactose (ELG) were transposed by ANSES (the French Agency for Food, Environmental and Occupational Health and Safety) [33] based on the threshold value of 100 g/day, established for adults in 2016: 4- to 7-year-olds: 60 g/day; 8- to 12-year-olds: 75 g/day; and 13- to 17-year-olds: 100 g/day. The WHO recommends that free sugars do not make up more than 10% of a child’s total energy intake (TEI) and suggests that there are additional benefits at 5%. Free sugars include mono- and disaccharides added by manufacturers, the people preparing the food or consumers themselves, as well as sugars that are naturally found in honey, syrups, fruit juices, and fruit juice concentrates [32].

#### 2.2.4. Degree of Food Processing

The French National Nutrition and Health Program (PNNS 4) 2019–2023 aims to stop the increased consumption of ultra-processed foods (UPFs) according to the NOVA classification, and reduce it by 20% in the French population, including children [30]. UPFs tend to be energy-dense; rich in free sugars, saturated fatty acids and sodium; and low in protein, dietary fibre, vitamins, minerals, and antioxidants. They are also highly palatable and with a high glycaemic load [34,35]. Consuming UPFs often seems correlated with the prevalence of overweight/obesity [36,37] and associated chronic diseases such as cancers, type 2 diabetes, and cardiovascular diseases [38,39].

The NOVA classification was used to categorise the foods included in the study into unprocessed or minimally processed (NOVA 1), processed (NOVA 3) and ultra-processed (NOVA 4) after analysing each component in the list of ingredients and noting the presence of markers: ultra-processed ingredients derived from extraction (gluten, lactose, caseins, etc.) and secondary processing (glucose-fructose syrup, hydrogenated fats, etc.); cosmetic additives (colourings, emulsifiers, melting salts, gelling and texturing agents, sweeteners, etc.) and flavourings (natural or otherwise) [35].

Some foods were categorised based on their preparation method (i.e., products that have the term “extruded” in the legal name, even if they do not contain UPF markers in the list of ingredients). Additives were identified in the list of ingredients based on Regulation (EU) No 1169/2011 and Regulation (EC) No 1333/2008.

### 2.3. Statistical Analysis

Categorical data were compared using Fisher’s exact test (significance level 5%), with a significant imbalance in the numbers. The test was performed using XLSTAT Sensory (version 2019.3.1).

## 3. Results

### 3.1. Characteristics of the Sample

A total of 1155 products were included in the study, including 3 food supplements, collected in 20 French stores, supermarkets, hypermarkets, and local markets. Half of the stores (50%; *n* = 10) are hypermarkets and supermarkets, 35% (*n* = 7) are organic food stores, and 15% (*n* = 3) are hard-discount retail chains. As regards the products, 70.22% (*n* = 811) are from hypermarkets and supermarkets, 18.53% (*n* = 214) from hard-discount retail chains, and 11.26% (*n* = 130) from organic food stores.

The products bear 143 different brands, mainly (63.03%; *n* = 728) private labels, while 36.97% (*n* = 427) of cases were national brands. Based on the legal name, they were divided into 23 consistent categories (Supplemental Table S1), established in a way that allows for an analysis using the various scoring systems. The main categories were biscuits, cakes and pastries (27.97%; *n* = 323), chocolate and sweets/candy (20.26%; *n* = 234), and dairy products (16.10%; *n* = 186).

A small proportion of the products included in the study come from organic farming (17.23%; *n* = 199) and were found mainly (65.33%) in organic food stores. The products in question are for the most part breakfast cereals (*n* = 50), fruit compotes (*n* = 42), and biscuits (*n* = 34). Additionally, nine categories are absent from our organic food stores pick up: pastries, soft drinks, ice cream, cheeses, flavoured milk, savoury snacks, flavoured water, fresh fruit, and breakfast biscuits.

### 3.2. Claims

Nutrition claims can be found on 411 products (35.58%), mainly related to sugar (12.29%), such as “no added sugar” and “low in sugar”, as well as calcium and vitamin D (11.17%). Health claims were found on 16 products (1.39%) and relate mainly to bone health. The sample also includes some claims relating to children, such as “developed by a team of paediatric nutritionists”, on 7 products (0.61%). The most common claims relate to ingredients (42.60% of products; *n* = 492), such as “no artificial colourings or flavourings”, which is the most frequent claim.

### 3.3. Marketing Methods

**Figure 1:**
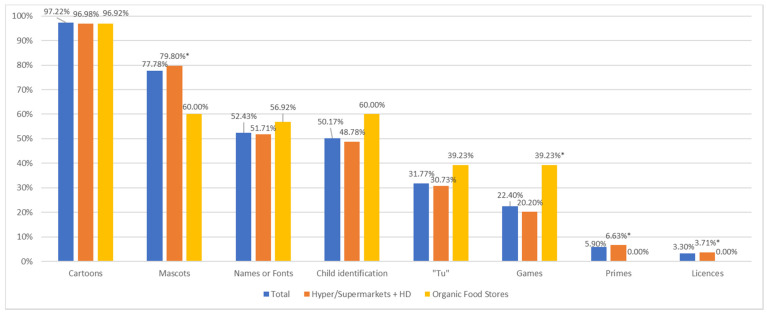
Overview of marketing strategies by store type. * significance risk 5%.

Cartoons and mascots are the most common hooks used in children’s marketing in our sample (*n* = 1155). In total, 97.22% of the products included in the study feature cartoons on the packaging (Figure 1). The practice can be observed to a similar extent in private labels (99.72%) and national brands (92.97%). Practices are relatively homogenous among the different store types. Nevertheless, mascots are significantly more often used in hypermarkets and supermarkets and hard-discount retail chains than in organic food stores, as are primes and licences, which are not used in organic food stores at all. Conversely, games are used significantly more often in products sold at organic food stores (Figure 1).

### 3.4. A Majority of Sugary Products

In total, 1081 products (93.60% of the sample) belong to the category of sweetened foods (naturally or through added sugar). They make up 17 of the 23 categories, including the first 9 (sweets/candy, biscuits, dairy desserts, breakfast cereals, fruit compotes, cakes, pastries, soft drinks, and ice cream) as well as chocolate, juices and nectars, chocolate powders, flavoured milk, cereal bars, flavoured water, breakfast biscuits, and fresh fruit.

The total sugar content has been recorded from the nutrition table for each product. Nutrition labelling do not specify the quantity of free sugars. Nevertheless, based on the lists of ingredients, it was possible to identify foods containing added sugars such as sugar, glucose syrup, glucose-fructose syrup, honey, invert sugar syrup, fructose, glucose, sugarcane molasses, and caramel syrup, as well as other sources of free sugars: concentrated and fresh fruit juices (excluding lemon and elderberry juice), in their ingredient lists, according to the WHO definition [32].

In total, 23.81% of the products in the sample (*n* = 275) list a sweetener as the first ingredient, which means that it is the main ingredient in the recipe. The products in question are mainly sweets/candy (*n* = 181), but also chocolate (*n* = 31), cakes (*n* = 28), biscuits (*n* = 22), and pastries (*n* = 10).

A large majority of products (89.52%; *n* = 1034) contain free sugars according to the WHO definition [32], mainly sugar (85.63% of products). In total, 46.23% (*n* = 534) of products contain ultra-processed sugars (glucose syrup, glucose-fructose syrup, etc.), 9.35% contain juices and juice concentrates, and 3.64% contain honey. Thirty-nine products contain 3 ultra-processed sugars: sugar, glucose syrup, and glucose-fructose syrup.

In total, 827 products (71.60%) suggest a recommended portion size, for instance in recommendation of complete snack or breakfast including the food item (contextualisation on pack). Among these, 124 products (10.74% of the sample) contain at least ¼ of the recommended sugar intake in 4- to 7-year-olds [33] in one portion (i.e., ≥15 g/portion), while 425 products (36.80% of the sample) contain at least 10 g of sugars per recommended portion size.

### 3.5. Added Salt: A Common Occurrence

In total, 43.98% of the products included in the study contain added salt, which applies to a large share of sweetened products: 100% of cereal bars and breakfast biscuits; more than 90% of biscuits (92.12%), cakes (97.96%), and pastries (98.33%); and 76.38% of breakfast cereals.

### 3.6. Displaying the Nutri-Score Label

Only 20.75% of products of the sample display their Nutri-Score group (*n* = 239). The practice is significantly more common in hyper/supermarkets and hard-discount retail chains (23.22% of products) than in organic food stores (0.79%, i.e., 1 product). In our sample, the categories that display the Nutri-Score group most often are flavoured milk (66.67%) and processed fish and nuggets (61.54%). Most products that bear a Nutri-Score label are in Nutri-Score groups D (31.38%). In total, 46.44% are in groups D and E (Table 1).

There are more products Nutri-Score A and B among products with a Nutri-Score label than among products without a Nutri-Score label, more of which are in Nutri-Score groups D and E (Table 1).

### 3.7. Nutri-Score Results of the Products in the Sample

Most (58.68%; *n* = 676) products included in the study are in Nutri-Score groups D and E, with the highest share in group D (39.32%; *n* = 453) Table 1.

The results are homogeneous for national brands and private labels identified in the 3 store types (results not shown), but there are significant differences between hyper/supermarkets/hard-retail discount retailers, and organic food stores with significantly more products Nutri-Score A and fewer products Nutri-Score D (Table 2).

**Figure 2:**
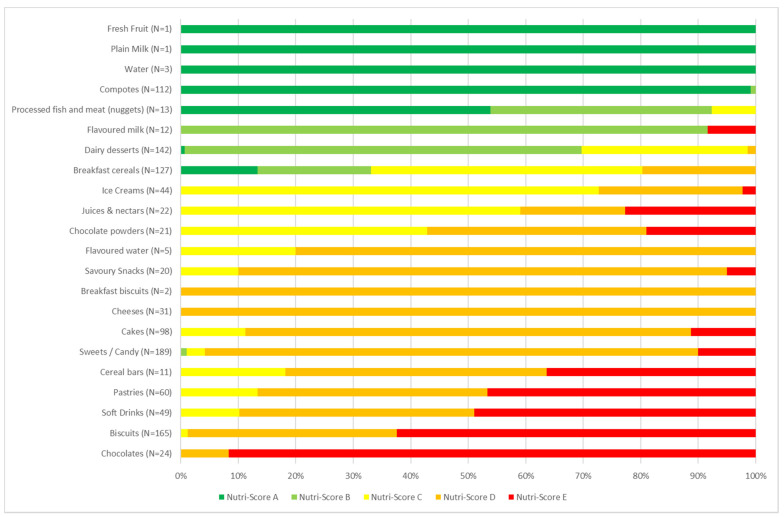
Graphical representation of the distribution of food groups in the sample (*n* = 22) by Nutri-Score group (*n* = 1152).

Four product categories are mainly (around 100%) Nutri-Score A: water, plain milk, fresh fruit, and compotes (Figure 2). More than half (53.85%) of processed fish and nuggets are Nutri-Score A. Most flavoured milks (91.67%) and dairy desserts (69.01%) are Nutri-Score B. Breakfast cereals (47.24%), ice creams (72.73%), and juices and nectars (59.09%) are mainly in group C. All cheeses and most cakes (77.55%), savoury snacks (85%), sweets/candy (85.71%), and flavoured waters (80%) are Nutri-Score D. Most pastries (46.67%), soft drinks (48.98%), biscuits (62.42%), and chocolates (91.67%) are Nutri-Score E (Supplemental Table S2).

In our sample, the correlation with the claims made shows that using nutrition claims has a decreasing gradient of Nutri-Score groups A to E. Health claims, which are rare in these children’s products, are mainly found on products in Nutri-Score group B, over-represented by dairy desserts: 100% of claims state that “calcium and vitamin D are necessary for normal growth and bone development in children”. Ingredient claims are distributed homogenously between Nutri-Score groups (Table 3).

### 3.8. Complementary Nutrition Labelling

A non-negligible percentage (35.85%; *n* = 413) of children’s products included in the study voluntarily specify how much one portion contributes to the reference dietary intakes of macronutrients for adults in the form of percentages in the nutrient table. The labelling is voluntary and provided for in regulations relating to the reference intakes of an average adult who consumes 2000 kcal (8400 kJ) [40]. For 66 products (66/1152; 5.73%), this information is included on the packaging together with the Nutri-Score group.

### 3.9. Compliance with the Criteria of the WHO Europe Nutrient Profile Model

In our sample of products marketed to children, 94.88% (*n* = 1152) do not meet the criteria of the WHO Europe Nutrient Profile Model [15] and would be therefore ineligible for marketing aimed at children (Table 4).

Most (57.37%), i.e., 627/1093 non-compliant products, belong to a category that is not authorised to use marketing aimed at children according to the WHO Europe Nutrient Profile Model, including all chocolates, sweets/candy, cereal bars, biscuits, cakes, pastries, fruit juices, and ice creams, without comparing the nutrient profile to threshold requirements (Table 4).

The second limiting criterion is the total sugar content: 336/1093 products (i.e., 30.74% of non-compliant products) exceed the upper limit for total sugars. The products in question include breakfast cereals, dairy products, fruit compotes (even without added sugars because the criterion is highly restrictive: 10 g/100 g). Limiting criterion number 3 is the presence of added sugars, which applies to 114 products, i.e., 10.43% of non-compliant products. Limiting criterion number 4 is fat content (8.6% of non-compliant products; *n* = 94) and applies to three categories: breakfast cereals, dairy products, and cheeses. The salt criterion applies to only 46/1093 (4.21% of non-compliant products) and relates to 3 categories: savoury snacks, cheeses, and dairy products. As the criterion is too flexible for the categories “processed meat, poultry, fish and similar” (>1.7 g/100 g) and “breakfast cereals” (>1.6 g/100 g) in our sample, all products in these categories comply with it. Conversely, the salt criterion is highly limiting for the “savoury snacks” category (>0.1 g/100 g) and 100% of products fail to meet it. Not relevant for the fruit compote category, all products in that category are compliant. The presence of non-sugar sweeteners is not limiting in our sample, which does not contain products with artificial sweeteners.

As such, 59 products meet WHO criteria. The highest compliance rates can be found in processed fish and nuggets, fresh fruit, plain milk, water, fruit compotes, and dairy desserts.

**Figure 3:**
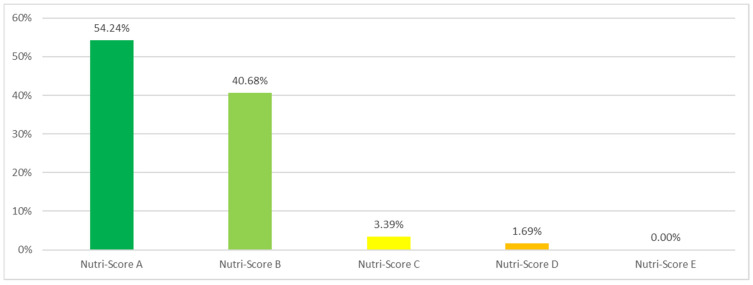
Distribution of Nutri-Score groups in products that meet the criteria of the WHO Europe Nutrient Profile Model (*n* = 59).

The share of organic foods in the 59 compliant products is higher than in the overall sample (*n* = 1152): 37.29% vs. 17.01%. The distribution of Nutri-Score groups is statistically different between the WHO compliant products and all the products included in the study: 94.92% of compliant products are in Nutri-Score groups A or B, i.e., four times more Nutri-Score groups A and three times more Nutri-Score groups B, which shows that the results are consistent as regards the Nutri-Score system and the WHO Europe Nutrient Profile Model (Figure 3).

Products that meet the criteria of the WHO Europe Nutrient Profile Model make twice as many nutrition claims (71.19% vs. 35.68% in the overall sample). The claims mainly relate to lower levels or absence of added sugars, and the presence of calcium and vitamin D.

More than half (54.24%) of products that meet the criteria of the WHO Europe Nutrient Profile Model are ultra-processed foods in NOVA group 4. The percentage is significantly lower in the overall sample (87.93%; *n* = 1152) and the percentage of minimally processed/unprocessed foods (NOVA group 1) is significantly higher (37.29% vs. 6.6%).

### 3.10. Degree of Processing

**Figure 4:**
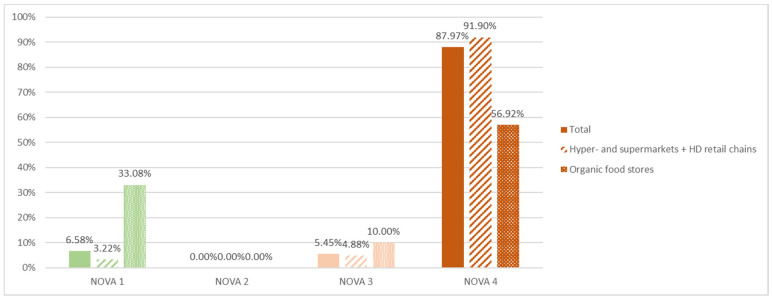
Distribution of NOVA groups for products included in the total sample (*n* = 1155) and by store type.

According to the NOVA classification, our sample (*n* = 1155) is mainly made up of ultra-processed foods in NOVA 4 (87.97%; *n* = 1016) and to a small extent of minimally processed/unprocessed products in NOVA group 1 (6.58%, *n* = 76) as well as processed products in NOVA 3 (5.45%; *n* = 63), and does not include any “processed culinary ingredients” in NOVA 2 (Figure 4). Products from organic food stores include significantly more minimally processed/unprocessed foods and fewer ultra-processed products (Figure 4), particularly because organic products in the sample include more minimally processed/unprocessed foods (26.13%) and fewer ultra-processed products (65.33%) than non-organic foods (NOVA 1: 2.51%; NOVA 4: 92.57%), and this is especially true for organic products from organic food stores (56.92% of UPFs vs. 81.16% for organic products from conventional stores).

Twelve categories include only ultra-processed foods: cereal bars, chocolates, flavoured milk and water, pastries, breakfast biscuits, cakes, ice cream, sweets/candy, soft drinks, and savoury snacks. The categories dairy desserts, breakfast cereals and biscuits are very similar (>90%). Three categories (plain milk, fresh fruit, and water) include only minimally processed or unprocessed foods (Supplemental Figure S1).

Analysing Nutri-Score groups based on NOVA groups shows that foods in Nutri-Score group A are significantly more often minimally processed/unprocessed (NOVA 1) and less ultra-processed (NOVA 4). From Nutri-Score group B (mainly dairy desserts), the rate of UPFs is very high (>90%) and relatively consistently between Nutri-Score groups B, C, D, and E, which suggests that children’s foods in general are often ultra-processed, regardless of their nutritional quality (Table 5).

An analysis of the ingredient lists showed that ultra-processed foods (NOVA 4; *n* = 1016) included in our sample have longer lists of ingredients, with an average number of 15.2 elements in the ingredient list, and a higher share of additives (3.28 on average) (Table 6).

Nevertheless, ultra-processed ingredients—and not additives—were the ones most often UPF markers found in the UPFs (*n* = 1016) included in our sample, such as flavourings and glucose syrup (Table 7).

Around one hundred other UPF markers were found in the foods and beverages included in our sample (results not shown).

In total, 95% of products in our sample that made at least one claim relating to “no artificial ingredients” or “natural ingredients” (*n* = 184), such as “100% natural ingredients”, “no artificial flavourings”, “no artificial colourings”, and “natural flavourings and colourings” are ultra-processed.

## 4. Discussion

This paper has for the first time analysed the nutritional quality of products marketed to children over 3 years old on the French market using the Nutri-Score labelling system, the WHO Europe Nutrient Profile Model and the NOVA classification as indicators, based on packaging information, from super- and hypermarkets, hard-discount retail chains, and organic food stores.

In our sample, the marketing means most often used to target children are cartoons (97.22%), as was observed in other studies [7,23,41,42], followed by mascots (77.78%), which are both known to influence children under 12 years old, especially as regards their choices, preferences, and eating habits [2,9,11].

Our results show that packaging marketed to children mainly relates to sugary foods, as has been pointed out by other authors [23], which is consistent with the fact that advertised products are most often sugar-based [20,43]. Almost a quarter of the products in our sample (23.81%; *n* = 275) list a sweetener as the first ingredient, which means that it is the main ingredient. A vast majority of products (89.52%; *n* = 1034) contain free sugars according to the WHO definition [32], mainly sugar (85.63%) and ultra-processed sugars (46.23%) such as glucose syrup and glucose-fructose syrup. These products (*n* = 1034) are mainly Nutri-Score D and E (62.08%; *n* = 640/1031, excluding food supplements), do not meet the criteria of the WHO Europe Nutrient Profile Model (97.77%; *n* = 1008/1031), and ultra-processed foods (93.13%; *n* = 963). In an analysis of 3427 European baby foods, Grammatikaki et al. [44] concluded also that foods with added sugars, free sugars, and any sweetener ingredient have a poorer nutritional profile.

Such foods are mainly intended for afternoon snack or for breakfast. They contribute to the excessive intake of total sugars (ELG) found by the ANSES based on the INCA2 study (the second French Individual and National Study on Food Consumption), exceeding the thresholds recommended for 75% of 4- to 7-year-olds and 60% of 8- to 12-year-olds, especially at afternoon break [33]. Similarly, based on the 2019 survey on food behaviour and consumption in France (CCAF 2019), the CREDOC consumer research centre found that 72.5% of 4- to 7-year-olds (*n* = 58) and 53.9% of 8- to 12-year-olds (*n* = 568) have a higher than recommended intake with children aged 3 to 17 years old (*n* = 1102) consume 86 g/day of total sugars (ELG) on average, while 86.8% have a free sugar intake that exceeds WHO recommendations (<10% Total Energy Intake), with 67 g per day on average [45].

After almost 3 years of implementation, in July 2020 the Nutri-Score label could be found on 50% of the volume of products sold in France [46]. In our sample of children’s products, only 20.75% (*n* = 239) of items bore a Nutri-Score label. Nevertheless, we identified national brands and private labels that are in the process of adopting the label on all their packaging, then this proportion could increase soon. A non-negligible percentage (35.85%) of the products included in our study voluntarily specify how much one portion contributes to the reference dietary intakes of macronutrients for adults, which raises questions about how relevant such information is for children’s products. For 66 products (66/1152; 5.73%), such information can be found on the packaging together with the Nutri-Score group on the front, which creates confusion.

In an assessment carried out 3 years after Nutri-Score was introduced, products that bear the Nutri-Score label are divided as follows: 31.7% A, 18.2% B, 19.6% C, 20.9% D, and 9.6% E [46]. In our sample, the percentage of products Nutri-Score D and E among products bearing a Nutri-Score label was higher (46.44%), which shows that children’s foods are more often in Nutri-Score groups D and E compared to foods in general. This is consistent with past findings, which suggest that children’s foods are richer in fat, sugar, and salt compared to products marketed to the general population [22]. Half (53.6%) of children’s food products included in an Australian study (*n* = 252) had a “less healthy” nutritional profile [25], for instance.

In our sample, products with a Nutri-Score label had more favourable scores than products without the label: slightly more were Nutri-Score A (14.23% vs. 11.72%) and fewer Nutri-Score D (31.38% vs. 41.4%) and E (15.06% vs. 20.48%). Similarly, in the Australian study, products that displayed a nutritional quality score (the Health Star Rating) were more often rated as “healthy” (73.8% vs. 59% in products without a score) [25].

Analysing the Nutri-Score of the products included in the study (*n* = 1152), with and without Nutri-Score label on pack, showed that most (58.68%; *n* = 676) children’s products are Nutri-Score D and E, with a majority in group D (39.32%; *n* = 453). The data are in line with food products in D and E advertised to children on television: 53.3% to 4- to 12-year-olds in 2018 according to the French Public Health Authority [11]; 88% according to the UFC-Que Choisir French consumers association between October and November 2019 [27]. The results are also consistent with other analyses of packaging on children’s products that were largely found to be “less healthy”, mainly due to their sugar levels, calculated at 77% according to the UK Ofcom nutrient profiling model [47] and 41% for the only selection of categories perceived and promoted as “healthy” by Garcia et al. [23]. Similarly, in Australia 62.2% of children’s products were “less healthy” [24] according to the Food Standards Australia New Zealand nutrient profiling scoring criterion. Children’s foods therefore tend to be mainly Nutri-Score D and E, which means they have a poor nutritional quality, and the French National Nutrition and Health Program (PNNS) recommends limiting their consumption [30].

All the products included in our study feature marketing elements targeting children (cartoons, mascots, games, prizes, informal forms of address) on the packaging. Yet 94.88% of products in the sample do not meet the criteria of the WHO Europe Nutrient Profile Model, which was designed to establish whether a product should be authorised to be marketed to children [15]. The result is higher than in the study by the European Commission (Joint Research Centre) on 2691 products available on the market in 2015 in 20 countries [48], which found 68% of non-compliance, but the authors had voluntarily entirely excluded certain categories from the analysis, such as chocolate, sweet biscuits, cakes and sugary drinks, which make up 57.37% (627/1093) of compliant products in our sample. As in our study, the criterion that was most often not met was total sugar intake, particularly due to breakfast cereals and yoghurts [48]. As such, sugar is definitely a cause for concern when it comes to food products marketed to children.

In a previous study by the European Consumer Organisation (BEUC), analysis of supermarket in 13 European countries found kids marketing (mascots and cartoon characters), on more than 100 food products, most of which did not meet the criteria of the WHO Europe Nutrient Profile Model [49]. The findings are consistent with advertisements aimed at children: 70% of products marketed to 4- to 12-year-olds in France do not meet WHO criteria [11].

Several studies have shown that children and adolescents are the main consumers of ultra-processed foods [50,51], with caloric intake mainly from UPFs: 67% in the US in 2018 in 2- to 19-year-olds [52] and 65.8% in the UK in 4- to 10-year-olds [53], even in Mediterranean based countries [54]. In France, analysis of data in the INCA3 study (1- to 10-year-olds; *n* = 1035) suggests that 45.5% of calories came from UPFs in 2014–2015, which is an increase compared to INCA1 (1998–1999; 42.8%) and INCA2 (2006–2007; 43.2%) [55]. In its call to action, the European Childhood Obesity Group warned about the negative effects of children consuming large amounts of UPFs and called for restrictions [56]. Several studies have shown that the percentage of UPFs in a diet is correlated to its quality, particularly in children [34,57,58]. For example, Martinez Steele et al. [57] have shown that there is a strong inverse linear relationship between the nutritional intake of UPFs on the one hand compared to that of protein, fibre, vitamins A, C, D and E, zinc, potassium, phosphorus, magnesium, and calcium, while the proportion of saturated fats and added sugars increases significantly due to UPFs. Some studies suggest that consuming a great deal of UPFs negatively affects the academic skills of children and adolescents [59]. A high intake of UPFs has therefore been associated with a higher body fat percentage [60], waist circumference [61], and BMI [62], as well as higher rates of dyslipidaemia [63,64] and metabolic syndrome [65] in children.

Characterised by the NOVA classification, our sample (*n* = 1155) is mainly made up of ultra-processed products (87.97%; *n* = 1016), and although it does not reflect a child’s total diet, it suggests a strong trend in food products marketed to children, with no less than 12 categories made up entirely of UPFs. Past studies on packaging (which were not focused on children’s products) found lower percentages of UPFs: 83% in supermarkets in New Zealand [66], 71% in the United States (for *n* = 230,156 foods and beverages) [67], 64.64% in France based on the collaborative database Open Food Facts [68], 67% [69] and 69% [70] using the SIGA method, which suggests that products marketed to children are highly ultra-processed. UPF markers are different for SIGA and NOVA (e.g., SIGA includes refined oils), but the two classifications nevertheless allow for a relevant comparison. As in our sample, UPFs have longer lists of ingredients: 13.2 components vs. 3.7 in minimally process/unprocessed foods [69], and 15.2 vs. 3.29 in NOVA group 1 and 5.89 in NOVA group 3 in our sample. The number of additives is also significantly higher in UPFs, as found in previous works [69,70]. The main UPF markers are not additives but flavourings, glucose syrup, and starch. It is therefore desirable to reduce additives as well as flavourings and ultra-processed sugars in children’s foods.

Although the results of the Nutri-Score labelling system and the WHO Europe Nutrient Profile Model are consistent (94.92% of products that meet WHO criteria are in Nutri-Score groups A and B), our study found that a significant percentage of foods in Nutri-Score groups B and C are nevertheless ultra-processed (96.48% and 93.26%), which suggests that Nutri-Score is an insufficient indicator for assessing the overall quality of food products.

Past studies have found a gradient depending on the Nutri-Score group, such as (*n* = 220,522) 8% of UPFs classed as Nutri-Score A, 13% classed as B, 23% classed as C, 31%c classed as D, and 25% classed as E [68], or in another study 23.9% of UPFs among products in Nutri-Score group A, 57.8% in Nutri-Score group B, 65.6% in Nutri-Score group C, 68% in Nutri-Score group D, and 85.6% in Nutri-Score group E [71]. In our sample, however, the percentage of UPFs is higher than 90% in foods in Nutri-Score groups B to E, which means that children’s foods are highly ultra-processed, regardless of their Nutri-Score.

According to Popkin et al. [72], food industry increasingly often adds micronutrients to UPFs formulations to be able to make certain nutrient and health claims. Our sample includes a large number of UPFs (*n* = 1016), yet the number of nutrition claims was relatively low (*n* = 411; 35.58%), while health claims were very rare (*n* = 16; 1.39%). Ingredient claims (*n* = 492; 42.6%) could be found on 43.5% of UPFs (442/1016) and 95% of products with claims such as “no artificial ingredients” and “natural ingredients” were ultra-processed in our sample. This percentage is much higher than the 31.4% found by the Joint Research Centre [44], but their study focused on baby food (*n* = 3427) and the products were found to be overall less ultra-processed (29.2%) because the related food regulation is stricter.

There are few organic foods marketed to children: they make up only 17.23% of our sample (*n* = 199/1155). Although they rarely feature the Nutri-Score label (0.79%, i.e., 1 product), organic products are significantly more often in Nutri-Score group A and less often in Nutri-Score group D. They are over-represented among foods that meet WHO criteria (37.29% vs. 17.01% in the overall sample) and they are less often ultra-processed (65.33% vs. 87.97% in the overall sample), particularly due to the high share of fruit compotes (*n* = 42), the lack of many categories found to be entirely ultra-processed such as pastries, soft drinks, savoury snacks, and ice cream, and the low percentage of sweets/candy and chocolate.

Half of the organic products included in the study were nevertheless ultra-processed, which is consistent with the results obtained using the SIGA classification (53%; *n* = 8554) [73], because although artificial flavourings are prohibited and fewer additives are authorised in organic farming, most UPF markers were ultra-processed ingredients such as glucose syrup. This is all the more true for organic foods found in conventional stores: 81.16% of UPFs vs. 56.92% in organic food stores. The same difference was observed in other studies [73,74], although our findings are higher than those of Desquilbet et al. [74], who recorded 31.4% and 26.8% respectively because their analysis included non-pre-packaged foods, i.e., raw foods. Our results are also higher than analyses performed using the SIGA classification [73], which found 56% and 48% of UPFs respectively even though the SIGA indicator includes more markers, which suggests that, even in the case of organic products, children’s foods are more often ultra-processed.

Lastly, among children’s foods, plain milk, fresh fruit, still water, and fruit compotes are items that seem most suitable in terms of the three indicators chosen: Nutri-Score, WHO Europe Nutrient Profile Model, and NOVA.

Our study has some limitations. Systematic data collection by a store operator could mean that relevant items were overlooked and not included. All food aisles (excluding baby food) were surveyed, however, which helped identify isolated items such as the only fresh fruit recorded. To limit the risk of error when transcribing information found on packaging, a different store operator performed systematic checks, followed by further checks for any modified data, which helped ensure that the data collected was accurate. Another possible limitation is the working group’s decisions about whether to include a product that could be intended for either children or adolescents; although the judgement call was always collective and unanimous, it could be seen as subjectivity bias.

As for sample size, the information collected allowed for a sufficient sample (*n* = 1155) to perform relevant analyses. For the Nutri-Score and WHO Europe Nutrient Profile Model classifications, categorising the items was relatively intuitive because all the products included in the study were quite traditional. Data found on packaging were always comprehensive, and official calculation tools and thresholds were used.

One of the study’s strong points is the manual analysis of the lists of ingredients to establish the degree of processing for each product according to the NOVA classification. The method ensured a level of accuracy that is not always found in studies, which often classify foods based only on their food category or name when the list of ingredients is unavailable [75,76]. Doing so groups food categories together without taking into account differences in formulations that help separate items into products in different NOVA groups, particularly in the case of biscuits and breakfast cereals. We consulted the research teams that developed the NOVA classification, thereby reducing the risk of error when identifying UPF markers that bring a food product down to NOVA group 4.

Our study is limited to pre-packaged foods available at supermarkets and featuring marketing elements aimed at children. The items included in our study do not reflect the total diet of children in France. Moreover, as in the case of other publications [44,48], we do not have consumer statistics for the products assessed, which means that it is not possible to establish direct correlations between their composition, the population’s nutritional intakes, overall diet, and health, although the fact that the products are available on the market suggests that they are consumed by the general population.

## 5. Conclusions

By analysing a number of food products in light of the Nutri-Score labelling system, the WHO Europe Nutrient Profile Model and the NOVA classification, our study suggests that a non-negligible share of pre-packaged foods marketed to children do not have a suitable nutrient profile. Various measures have been considered to limit marketing pressure on children to products with a high nutritional quality, including legislation proposed in France [77], proposals to amend EU regulations [78], local Advertising Code [4], and voluntary initiatives by leading food and beverage companies (EU Pledge). The measures relate to television and Internet exposure, but they do not always cover packaging even though the latter is an important vector of influence, and they rarely include criteria relating to the degree of processing. As such, marketing elements aimed at children should no longer appear on ultra-processed products, products in Nutri-Score group D and E, and products that do not meet the criteria of the WHO Europe Nutrient Profile Model, as advocated by the European Consumer Organisation (BEUC), which since 2017 has campaigned to stop using marketing techniques aimed at children (brand mascots and cartoon characters) on the packaging of unhealthy foods [49]. The BEUC reiterated its call to action in 2021 [79].

The market should be analysed regularly to ensure that rapid developments are taken into account and to monitor not only how marketing practices change, but also how the nutritional quality of products is affected. The analysis should also be extended to other European countries.

## Figures and Tables

**Table 1:** Link between Nutri-Score group and voluntary display (*n* = 1152).

	Nutri-Score Label		No Nutri-Score Label		All Products (with/without Nutri-Score Label)	
	# of Products	% of Total	# of Products	% of Total	# of Products	% of Total
Nutri-Score A	34	14.23%	107	11.72%	141	12.24%
Nutri-Score B	50	20.92%	92	10.08%	142	12.33%
Nutri-Score C	44	18.41%	149	16.32%	193	16.75%
Nutri-Score D	75	31.38%	378	41.40%	453	39.32%
Nutri-Score E	36	15.06%	187	20.48%	223	19.36%
TOTAL	239		913		1152	

**Table 2:** Breakdown of Nutri-Score group by store type.

	Total		Hyper- and Supermarkets + HD Retail Chains		Organic Food Stores	
	# of Products	% of Total	# of Products	% of Total	# of Products	% of Total
Nutri-Score A	141	12.24%	95	9.27%	46	36.22%
Nutri-Score B	142	12.33%	132	12.88%	10	7.87%
Nutri-Score C	193	16.75%	172	16.78%	21	16.54%
Nutri-Score D	453	39.32%	423	41.27%	30	23.62%
Nutri-Score E	223	19.36%	203	19.80%	20	15.75%
TOTAL	1152		1025		127	

**Table 3:** Distribution of claims by Nutri-Score group.

	Total	Nutrition Claims		Health Claims		Ingredient Claims		Child-Related Claims	
		# of Products	% of Total	# of Products	% of Total	# of Products	% of Total	# of Products	% of Total
Nutri-Score A	141	126	89.36%	0	0.00%	47	33.33%	4	2.84%
Nutri-Score B	142	110	77.46%	9	6.34%	76	53.52%	0	0.00%
Nutri-Score C	193	80	41.45%	1	0.52%	89	46.11%	2	1.04%
Nutri-Score D	453	73	16.11%	6	1.32%	201	44.37%	0	0.00%
Nutri-Score E	223	22	9.87%	0	0.00%	79	35.43%	1	0.45%
	1152	411		16		492		7	

**Table 4:** Overview of compliance with the criteria of the WHO Europe Nutrient Profile Model [15] by food category.

Category	TOTAL	Non-Compliant		Compliant	
		# of Products	% of Total	# of Products	% of Total
Chocolate	45	45	100%	0	0%
Sweets/candy	189	189	100%	0	0%
Cereal bars	11	11	100%	0	0%
Cakes	98	98	100%	0	0%
Biscuits	167	167	100%	0	0%
Pastries	60	60	100%	0	0%
100% fruit juice	13	13	100%	0	0%
Ice cream	44	44	100%	0	0%
Savoury snacks	20	20	100%	0	0%
Fresh fruit	1	0	0%	1	100%
Fruit Compotes	112	94	83.03%	18	16.97%
Plain milk	13	12	92.3%	1	7.7%
Processed fish and meat (nuggets)	13	0	0%	13	100%
Breakfast cereals	127	121	95.28%	6	4.72%
Other beverages	66	63	95.45%	3	4.55%
Cheeses	31	30	96.77%	1	3.23%
Dairy desserts	142	126	88.73%	16	11.27%
Total	1152	1093	94.88%	59	5.12%

**Table 5:** Percentage of products in NOVA groups 1, 2 and 4 by Nutri-Score group in the overall sample (*n* = 1155).

	Nutri-Score A	Nutri-Score B	Nutri-Score C	Nutri-Score D	Nutri-Score E
NOVA 1	62 (43.97%)	1 (0.70%)	9 (4.66%)	2 (0.44%)	2 (0.90%)
NOVA 3	39 (27.66%)	4 (2.82%)	4 (2.07%)	8 (1.77%)	9 (4.04%)
NOVA 4	40 (28.37%)	137 (96.48%)	180 (93.26%)	443 (97.79%)	212 (95.07%)

**Table 6:** Number of elements and number of additives in the list of ingredients by degree of processing as defined in the NOVA classification (*n* = 1155).

	Number of Elements in the Ingredient Lists				Number of Additives			
	Mean	Minimum	Maximum	Median	Mean	Minimum	Maximum	Median
NOVA 1 [*n* = 76]	3.29	1	8	3	0.42	0	2	0
NOVA 3 [*n* = 63]	5.89	3	16	5	0.77	0	2	0
NOVA 4 [*n* = 1016]	15.2	1	42	15	3.28	0	18	3

**Table 7:** Main UPF markers found in ultra-processed foods.

	UPF Markers	Status	Number of Products Containing Them	Presence in Ultra-Processed Products (*n* = 1016)
1	Natural flavourings	Non-additive	509	50.25%
2	Glucose syrup	Non-additive	360	35.54%
3	Artificial flavourings	Non-additive	333	32.87%
4	Lecithins	Additive	330	32.58%
5	Starch	Non-additive	282	27.84%
6	Gelatine	Non-additive	143	14.12%
7	Glucose-fructose syrup	Non-additive	137	13.52%
8	Dextrose	Non-additive	127	12.54%
9	Modified starch	Additive	95	9.38%
10	Maltodextrin	Non-additive	36	3.55%

## Data Availability

Data sets generated during the study are available from the corresponding author on reasonable request.

## Supplementary Materials

The following supporting information can be downloaded at: https://www.mdpi.com/article/10.3390/nu14010171/s1, Table S1. Product breakdown by category and store type (*n* = 1155), Table S2. Distribution of food groups in the sample (*n* = 22) according to the Nutri-Score labelling system, Figure S1. Distribution of food groups in the sample (*n* = 1155; 23 food groups) according to the NOVA classification.
