# Supplementary material for: Nutritional Quality and Degree of Processing of Children’s Foods Assessment on the French Market
Source: Nutrients. 2021 Dec 30;14(1):171. doi: 10.3390/nu14010171 (PMC8747148; doi:10.3390/nu14010171)
Supplement: Supplementary file 1 [file nutrients-14-00171-s001.zip › Supplemental Figure S1, Tables S1 and S2.pdf]

Supplementary Materials

Figure S1. Distribution of food groups in the sample (*n* = 1 155 ; 23 food groups) according to the NOVA classification

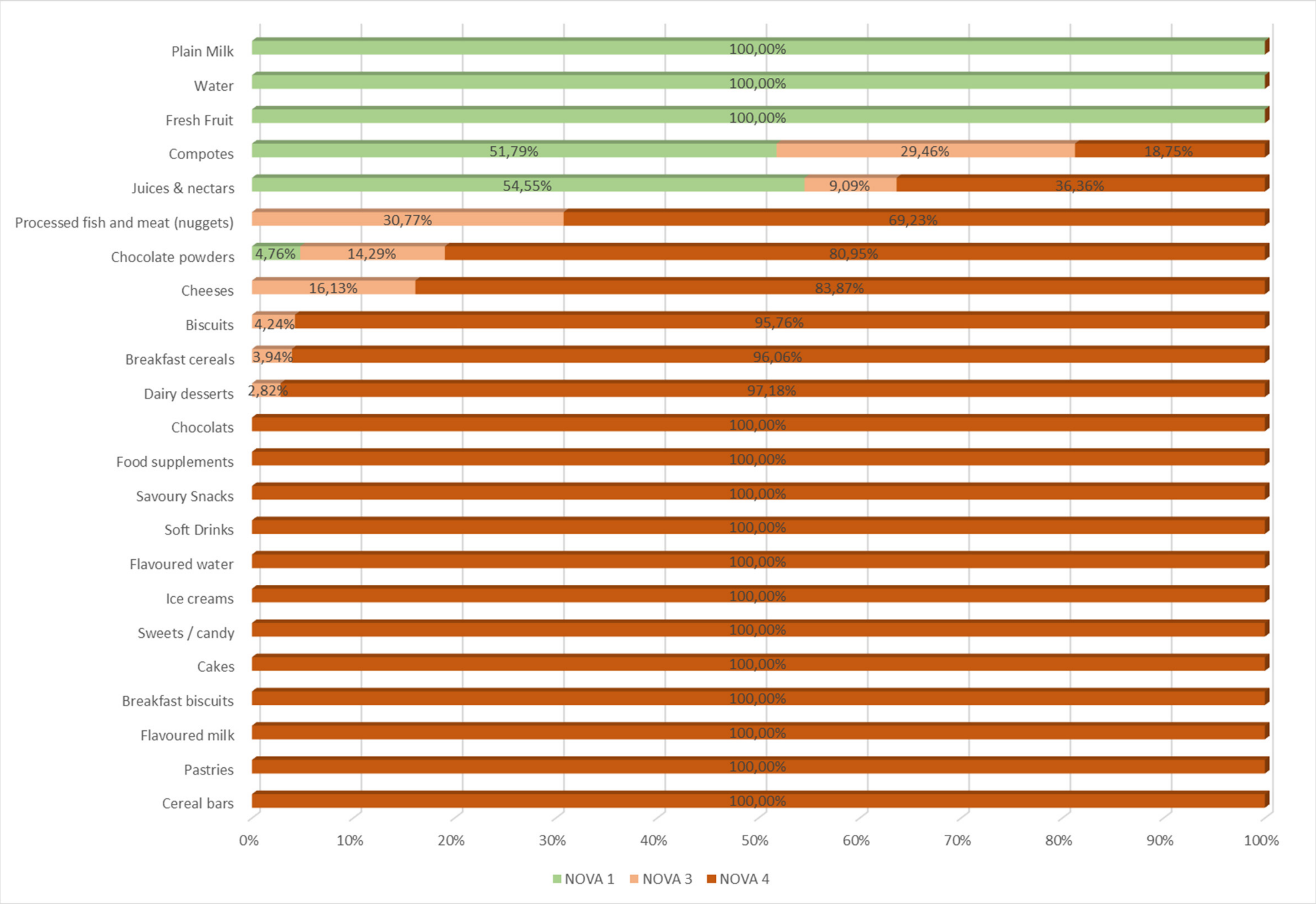

Supplementary Materials

Table S1. Product breakdown by category and store type (*n* = 1 155)

Categories	General		Super & Hypermarkets		Hard Discount (HD)		Super & Hypermarkets + HD		Organic Food stores	
Sweets / Candy	189	16.48%	150	20.75%	33	15.42%	183	17.85%	6	4.62%
Biscuits	165	14.39%	101	13.97%	41	19.16%	142	13.85%	23	17.69%
Dairy desserts	142	12.38%	109	15.08%	28	13.08%	137	13.37%	5	3.85%
Breakfast cereals	127	11.07%	71	9.82%	25	11.68%	96	9.37%	31	23.85%
Compotes	112	9.76%	56	7.75%	20	9.35%	76	7.41%	36	27.69%
Cakes	98	8.54%	80	11.07%	12	5.61%	92	8.98%	6	4.62%
Pastries	60	5.23%	51	7.05%	9	4.21%	60	5.85%	0	0.00%
Soft Drinks	49	4.27%	43	5.95%	6	2.80%	49	4.78%	0	0.00%
Ice creams	44	3.84%	40	5.53%	4	1.87%	44	4.29%	0	0.00%
Cheeses	31	2.70%	20	2.77%	11	5.14%	31	3.02%	0	0.00%
Chocolates	24	2.09%	10	1.38%	8	3.74%	18	1.76%	6	4.62%
Juices & nectars	22	1.92%	16	2.21%	0	0.00%	16	1.56%	6	4.62%
Chocolate powders	21	1.83%	13	1.80%	3	1.40%	16	1.56%	5	3.85%
Savoury Snacks	20	1.74%	15	2.07%	5	2.34%	20	1.95%	0	0.00%
Processed fish and meat (nuggets)	13	1.13%	8	1.11%	4	1.87%	12	1.17%	1	0.77%
Flavoured milk	12	1.05%	11	1.52%	1	0.47%	12	1.17%	0	0.00%
Cereal bars	11	0.96%	6	0.83%	4	1.87%	10	0.98%	1	0.77%
Flavoured water	5	0.44%	5	0.69%	0	0.00%	5	0.49%	0	0.00%
Food supplements	3	0.26%	0	0.00%	0	0.00%	0	0.00%	3	2.31%
Water	3	0.26%	3	0.41%	0	0.00%	3	0.29%	0	0.00%
Breakfast biscuits	2	0.17%	2	0.28%	0	0.00%	2	0.20%	0	0.00%
Milk	1	0.09%	0	0.00%	0	0.00%	0	0.00%	1	0.77%
Fresh fruit	1	0.09%	1	0.14%	0	0.00%	1	0.10%	0	0.00%
**TOTAL**	**1155**		**811**		**214**		**1025**		**130**	

Supplementary Materials

Table S2. Distribution of food groups in the sample (*n* = 22) according to the Nutri-Score labelling system

	Nutri-Score A	Nutri-Score B	Nutri-Score C	Nutri-Score D	Nutri-Score E
Sweets / Candy	0 (0.00%)	2 (1.06%)	6 (3.17%)	162 (85.71%)	19 (10.05%)
Biscuits	0 (0.00%)	0 (0.00%)	2 (1.21%)	60 (36.36%)	103 (62.42%)
Dairy desserts	1 (0.70%)	98 (69.01%)	41 (28.87%)	2 (1.41%)	0 (0.00%)
Breakfast cereals	17 (13.39%)	25 (19.69%)	60 (47.24%)	25 (19.69%)	0 (0.00%)
Compotes	111 (99.11%)	1 (0.89%)	0 (0.00%)	0 (0.00%)	0 (0.00%)
Cakes	0 (0.00%)	0 (0.00%)	11 (11.22%)	76 (77.55%)	11 (11.22%)
Pastries	0 (0.00%)	0 (0.00%)	8 (13.33%)	24 (40.00%)	28 (46.67%)
Soft Drinks	0 (0.00%)	0 (0.00%)	5 (10.20%)	20 (40.82%)	24 (48.98%)
Ice creams	0 (0.00%)	0 (0.00%)	32 (72.73%)	11 (25.00%)	1 (2.27%)
Cheeses	0 (0.00%)	0 (0.00%)	0 (0.00%)	31 (100.00%)	0 (0.00%)
Chocolates	0 (0.00%)	0 (0.00%)	0 (0.00%)	2 (8.33%)	22 (91.67%)
Juices & nectars	0 (0.00%)	0 (0.00%)	13 (59.09%)	4 (18.18%)	5 (22.73%)
Chocolate powders	0 (0.00%)	0 (0.00%)	9 (42.86%)	8 (38.10%)	4 (19.05%)
Savoury Snacks	0 (0.00%)	0 (0.00%)	2 (10.00%)	17 (85.00%)	1 (5.00%)
Processed fish and meat (nuggets)	7 (53.85%)	5 (38.46%)	1 (7.69%)	0 (0.00%)	0 (0.00%)
Flavoured milk	0 (0.00%)	11 (91.67%)	0 (0.00%)	0 (0.00%)	1 (8.33%)
Cereal bars	0 (0.00%)	0 (0.00%)	2 (18.18%)	5 (45.45%)	4 (36.36%)
Flavoured water	0 (0.00%)	0 (0.00%)	1 (20.00%)	4 (80.00%)	0 (0.00%)
Water	3 (100.00%)	0 (0.00%)	0 (0.00%)	0 (0.00%)	0 (0.00%)
Breakfast biscuits	0 (0.00%)	0 (0.00%)	0 (0.00%)	2 (100.00%)	0 (0.00%)
Plain Milk	1 (100.00%)	0 (0.00%)	0 (0.00%)	0 (0.00%)	0 (0.00%)
Fresh fruit	1 (100.00%)	0 (0.00%)	0 (0.00%)	0 (0.00%)	0 (0.00%)
Total	141	142	193	453	223
